# Supplementary material for: Risk Factors Associated with Diarrheal Episodes in an Agricultural Community in Nam Dinh Province, Vietnam: A Prospective Cohort Study
Source: Int J Environ Res Public Health. 2022 Feb 21;19(4):2456. doi: 10.3390/ijerph19042456 (PMC8872501; doi:10.3390/ijerph19042456)
Supplement: Supplementary file 1 [file ijerph-19-02456-s001.zip › ijerph-1550510-supplementary.pdf]

Figure S1. Questionnaire for socioeconomic status

CENSUS FORM, 2014

Census form number:[__ __ __ __ __ __ __]

A.1.a. Hamlet:_______________________________ 1.b. Group.:________________________

A.2 Detailed address:______________________________________ Phone number_______________ GIS ID number:[________]

A.3. Respondent's name:___________________________________ Member number of the respondent:☐☐

If this form was not filled in, what was the reason?____☐☐

No.	FULL NAME	Demo Status	SEX 1:M 2: F	Age in years	DOB dd/mm/yyyy	Rel. to HH	Fa ID	Mo ID	Health Insurance Card NO.	Mar Status	Edu Level	Present medical illness
01					___/___/_______							
02					___/___/_______							
03					___/___/_______							
04					___/___/_______							
05					___/___/_______							
06					___/___/_______							
07					___/___/_______							
08					___/___/_______							
09					___/___/_______							
10					___/___/_______							
11					___/___/_______							
12					___/___/_______							
13					___/___/_______							
14					___/___/_______							
15					___/___/_______							

___/___/______

Socioeconomic Status

Please circle the answers

1. Is this dwelling a single house or an apartment?
   1= Single house 2= Apartment

2. Who owns your house or your apartment?
   1= Household head 2= Other person (rented house/apartment)

3. What is the <u>main</u> composition of the walls of the dwelling?
   1= Cement, 2= Bricks, 3= Wooden Planks,
   4= Mud bricks, 5= Tin, 6= Sticks
   8= Other (specify) _______________

4. How large is your house (in square meters)? _______ m^2
   (including corridors, stairs, kitchens,…. UUU if unknown)

5. What is the water source in your house (multiple choices are allowed)?
   1=Tap, 2=Water Truck, 3= Tube well / hand pump,
   4=Open well, 5=Rain Water 6=Canal/River
   7=Lake/Pond 8=Other (specify) _______________

6. What is the source of drinking water in your house?
   1=Tap, 2=Water Truck, 3= Tube well / hand pump,
   4=Open well, 5=Rain Water 6=Canal/River
   7=Lake/Pond 8=Bottle, mineral water 9=Other, specify_________________________

7. Do you boil water before drinking? 1=Yes 2= No

8. What kind of toilet do you have? 1=Flush toilet, 2=Pit latrine, 9=Other, specify________________

9. Please tick all items owned by the family:
   ☐ Radio ☐ TV ☐ VCR ☐ Fan
   ☐ Refrigerator ☐ Washing M/c ☐ Sewing M/c ☐ Air conditioner
   ☐ Bicycle ☐ Motorcycle ☐ Car/Truck ☐ Shop
   ☐ Telephone ☐ Mobile phone ☐ Computer ☐ Internet
   ☐ Small Machine for Farming ☐ Large Machine for Farming
   ☐ Others (specify) ____
   ☐ **Does not have any items in the list**

Census form number:[__ __ __ __ __ __ __]

10. In the household, who smokes cigarettes /tobacco? (*write member No. and* ✓☐)

Member No.	Cigarettes	Tobacco	Smoke indoors?
__ __	☐	☐	☐
__ __	☐	☐	☐
__ __	☐	☐	☐
__ __	☐	☐	☐
__ __	☐	☐	☐

11. In the household, who drink alcohol, and how often? (*write member no and* ✓☐)

Member No.	Everyday	Sometimes	Once in a while
__ __	☐	☐	☐
__ __	☐	☐	☐
__ __	☐	☐	☐
__ __	☐	☐	☐
__ __	☐	☐	☐

12. In your household compound, How many following animals or pets are there? ☐ No animal
    [____] Pigs [____] Buffalos [____] Dogs
    [____] Cows [____] Cats [____] Chickens or birds
    [____] Ducks/Geese [____]Others: ___________

13. If anyone has chronic disease, please specify the following :
    ☐ No one has chronic disease

Member No	Full Name	a. Chronic disease	b. Commonly used drugs
__ __			
__ __			
__ __			
__ __			
__ __			

	Full Name	MS	Date
Enumerator			__/__/2006
Reviewer:			__/__/2006

	Full Name	MS	date
1st data entry			__/__/2006
2nd data entry:			__/__/2006

CODE LIST:
Demo status: 1=Present; 2= Migrated/death/duplicated
Relationship to HH:

1=Head of the family	7=Spouse of the family head
2=Son/Daughter of the family head	8=Spouse of the Son/Daughter of the family head
3=Grandchild of the family head	9=Spouse of the Grandchild of the family head
4=Brother/Sister of the family head	10=Spouse of the Brother/Sister of the family head
5=Mother/Father of the family head	11=Mother/Father in law of the family head
6=Extended Family (child of the brother/sister of HH, Grandparent of HH)	12=Others (cousin of the spouse of HH, relative of spouse of HH, person who rent the house)

Father's ID: If father's name is listed on census form of the household, please copy the cardinal number of the father and fill in this column.

Mother's ID: If mother's name is listed on the census form of the household, please copy the cardinal number of the mother and fill in this column.

Marital status:
1=Single
2=Married
3=Divorced / separated
4=Widowed
5=Others

Education level:
0=No school / Illiterate
1 ~ 12 = Finished classes (grade)
13=Finish primary college
14=Intermediate college
15=Higher college
16=University or post graduated

Occupation:

**1=Professional**<br>**2=Office worker**<br>**3=Owner of business premise**<br>**4=Uniformed worker**<br>**5=Unskilled manual worker**<br>**6=Skilled manual worker**<br>**7=Food seller (on the street)**<br>**8=Street seller**<br>**9=Farmer**<br>**10=Cook**	**11=Driver**<br>**12=Soldier**<br>**13=Student**<br>**14=Unemployed**<br>**15=Retired**<br>**16=Housewife**<br>**17=Garbage collector, Sewage work**<br>**18=Small business**<br>**19=Fishery**<br>**20=Children<age6**<br>**21=Other**

Khanh Hoa Health Project: Census and Socioeconomic Status

Present Medical Illness:

**1=Tuberculosis**<br>**2=Malaria**<br>**3=Leprosy**<br>**4=Goiter**<br>**5=Mental disorder**<br>**6=Physical disability**<br>**7=Meningitis/Encephalitis**<br>**8=Polio**<br>**9=Diabetes mellitus**	**10=Hypertension**<br>**11=Heart failure**<br>**12=Cleft lip/palate**<br>**13=Depression**<br>**14=Hepatitis**<br>**15=Measles**<br>**16=Mumps**<br>**17=Congenital heart disease**<br>**18=Other**<br>**88=No medical illness**<br>**99=Unknown**

Figure S2. Distribution of the study population according to the distance from WPP and diarrhea episode

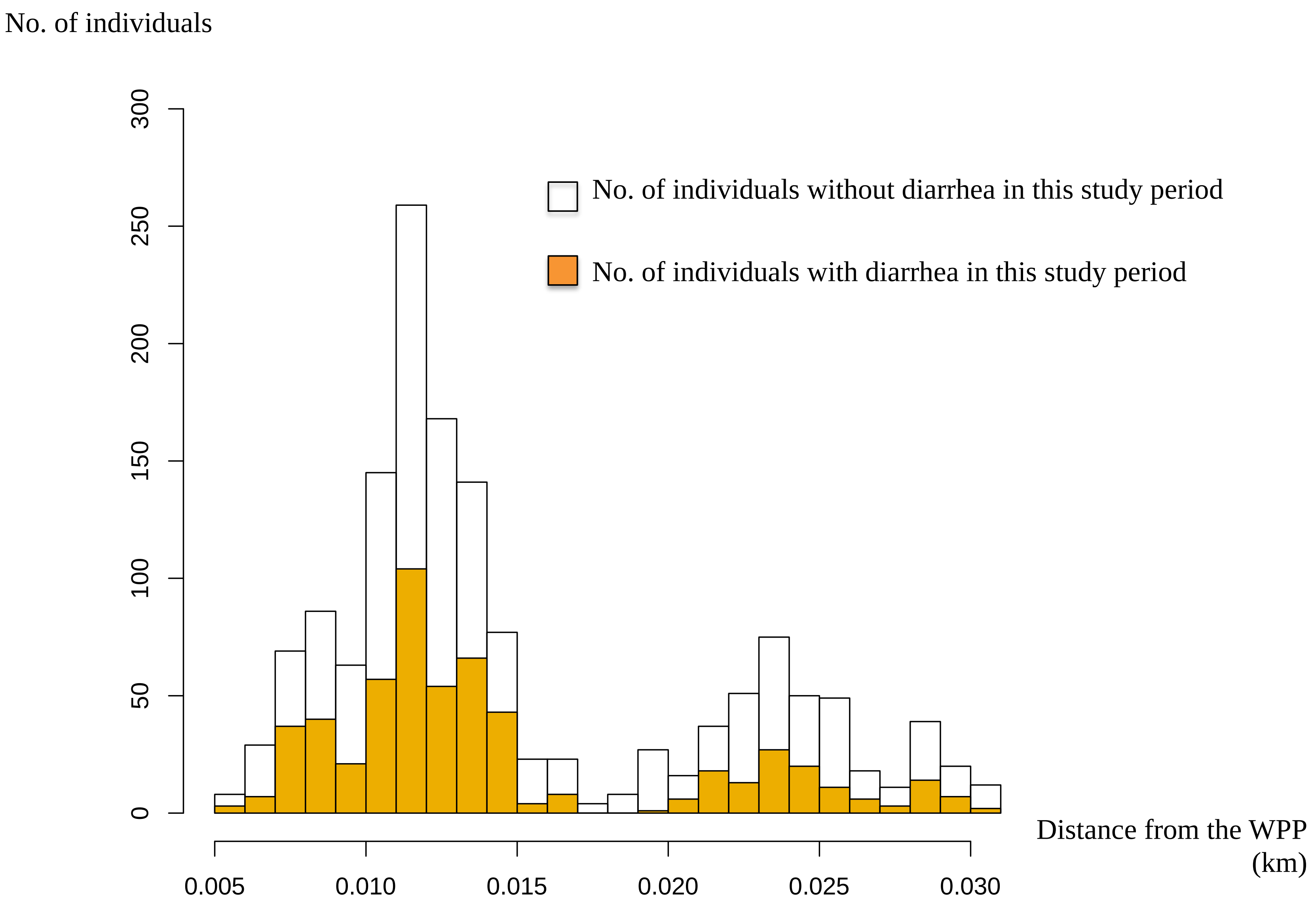

Figure S3. Age pyramid of the study population under 5 years old children and diarrhea episode (same as **Additional file 4: Table S2**)

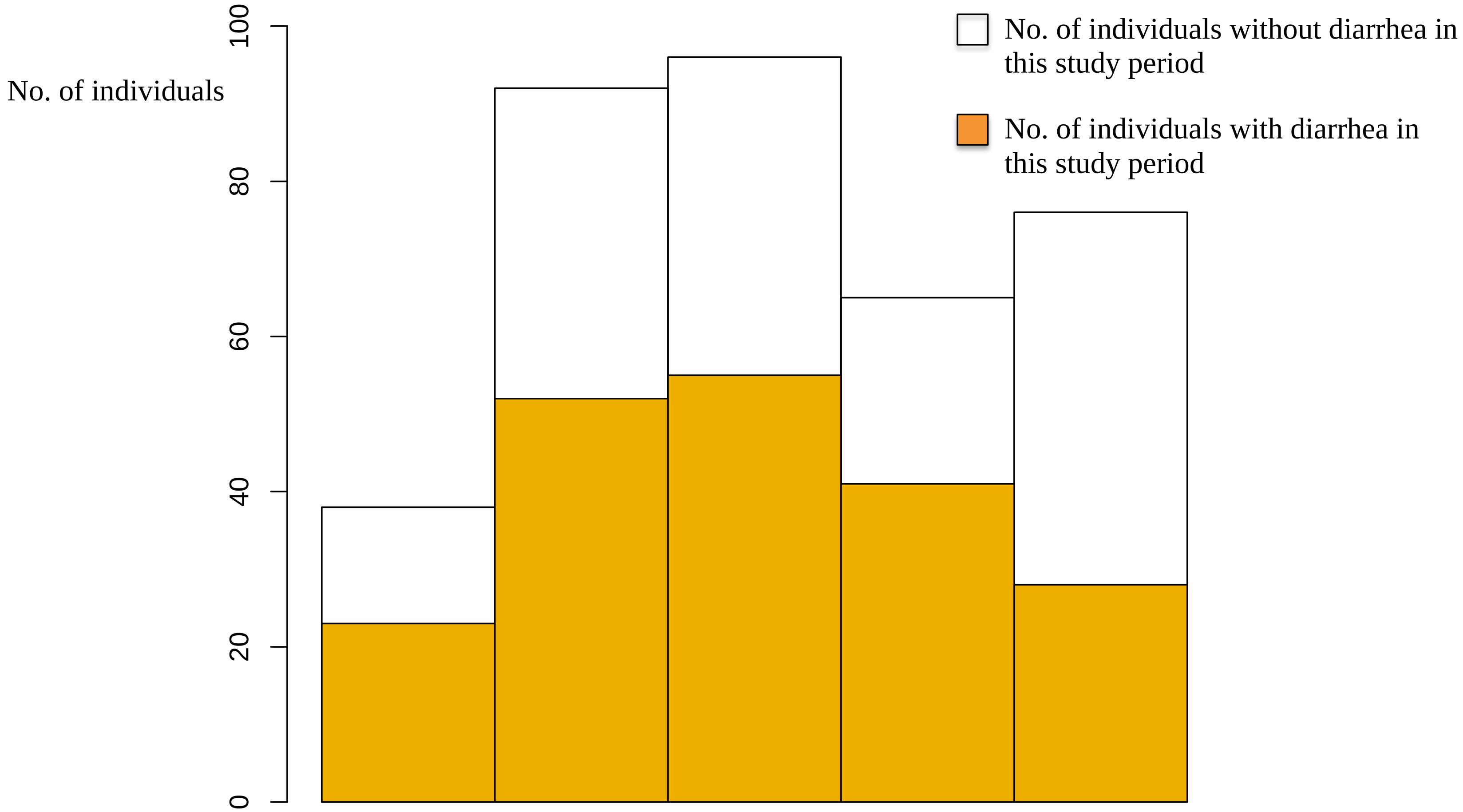

Age (years)	0	1	2	3	4
No. without diarrhea	15 (39.5%)	40 (43.5%)	41 (42.7%)	24 (36.9%)	48 (63.2%)
**No. with diarrhea**	**23 (60.5%)**	**52 (56.5%)**	**55 (57.3%)**	**41 (63.1%)**	**28 (36.8%)**
No. of individuals	38	92	96	65	76

Table S1 Ownership of household assets

Household asset	Ownership (n=311)		
	Yes	No	% of ownership
Radio	36	275	11.6
TV	299	12	96.1
VCR (Video Cassette Recorder)	176	135	56.6
Fan	306	5	98.4
Refrigerator	214	97	68.8
Washing Machine	72	239	23.2
Sewing Machine	66	245	21.2
Air conditioner	16	295	5.1
Bicycle	284	27	91.3
Motorcycle	293	18	94.2
Car/Truck	4	307	1.3
Telephone	28	283	9
Mobile phone	256	55	82.3
Computer	31	280	10
Internet	16	295	5.1

Table S2. Distribution of diarrheal episodes of per year of age under 5-year-old

	Individuals without diarrhea	Individuals with diarrhea	Cumulative episodes of diarrhea	Person-years of observation	Diarrhea episodes/person per year (95%CI)
under 1	15	23	41	39.35	1.04 (0.67-1.41)
1_<2	40	52	82	95.28	0.86 (0.67-1.06)
2_<3	41	55	83	99.42	0.83 (0.61-1.06)
3_<4	24	41	55	67.32	0.82 (0.62-1.01)
4_<5	48	28	47	78.71	0.60 (0.40-0.82)

Table S3. Model selection of multivariate NB-GLMM (Model 1)

Each row presents the explanatory variables for each model. Household was the random effects. The model with the lowest AIC is shown in boldface italic type

Explanatory variables	AIC	ΔAIC
Sex, Age, Density, Tap water, Water truck, Tube well/Hand pump, Open well, Rain water, Lake/Pond, Boil water, Distance from WPP, Toilet, Pig, Buffalos, Dogs, Cattle, Cats, Chicken, Ducks, Existance of animals, Wealth index	2703.2	19.2
Sex, Age, Density, Tap water, Water truck, Tube well/Hand pump, Open well, Rain water, Lake/Pond, Boil water, Distance from WPP, Toilet, Pig, Buffalos, Dogs, Cattle, Cats, Chicken, Ducks, Existance of animals	2699.6	15.6
Sex, Age, Density, Tap water, Water truck, Tube well/Hand pump, Open well, Rain water, Lake/Pond, Boil water, Distance from WPP, Toilet, Pig, Buffalos, Dogs, Cats, Ducks	2693.7	9.7
Sex, Age, Density, Tap water, Water truck, Tube well/Hand pump, Open well, Rain water, Lake/Pond, Boil water, Distance from WPP, Toilet, Pig, Dogs, Cats, Ducks	2691.7	7.7
Sex, Age, Density, Tap water, Water truck, Tube well/Hand pump, Open well, Rain water, Lake/Pond, Boil water, Distance from WPP, Toilet, Pig, Dogs, Cats	2690.1	6.1
Sex, Age, Density, Tap water, Water truck, Tube well/Hand pump, Open well, Rain water, Lake/Pond, Boil water, Distance from WPP, Toilet, Pigs, Cats	2688.2	4.2
Sex, Age, Density, Tap water, Water truck, Tube well/Hand pump, Open well, Rain water, Lake/Pond, Boil water, Distance from WPP, Toilet, Cats	2686.9	2.9
Sex, Age, Density, Tap water, Tube well/Hand pump, Open well, Rain water, Lake/Pond, Boil water, Distance from WPP, Toilet, Cats	2685.7	1.7
Age, Density, Tap water, Tube well/Hand pump, Open well, Rain water, Lake/Pond, Boil water, Distance from WPP, Toilet, Cats	2685.4	1.4
Age, Tap water, Tube well/Hand pump, Open well, Rain water, Lake/Pond, Boil water, Distance from WPP, Toilet, Cats	2685.2	1.2
Age, Tap water, Tube well/Hand pump, Open well, Rain water, Boil water, Distance from WPP, Toilet, Cats	2684.9	0.9
Age, Tap water, Tube well/Hand pump, Rain water, Boil water, Distance from WPP, Toilet, Cats	***2684.0***	***0***

Table S4. Model selection of multivariate NB-GLMM (Model 2)

Each row presents the explanatory variables for each model. Household was the random effects. The model with the lowest AIC is shown in boldface italic type

Explanatory variables	AIC	ΔAIC
Sex, Age, Density, Tap water, Water truck, Tube well/Hand pump, Open well, Lake/Pond, Drinking water, Boil water, Distance from WPP, Toilet, Pig, Buffalos, Dogs, Cattle, Cats, Chicken, Ducks, Existance of animals, Wealth index	2704.2	19.5
Sex, Age, Density, Tap water, Water truck, Tube well/Hand pump, Open well, Lake/Pond, Drinking water, Boil water, Distance from WPP, Toilet, Pig, Buffalos, Dogs, Cattle, Cats, Chicken, Ducks, Existance of animals	2700.6	15.9
Sex, Age, Density, Tap water, Water truck, Tube well/Hand pump, Open well, Lake/Pond, Drinking water, Boil water, Distance from WPP, Toilet, Pig, Buffalos, Cats, Chicken, Ducks	2694.7	10.0
Sex, Age, Density, Tap water, Water truck, Tube well/Hand pump, Open well, Lake/Pond, Drinking water, Boil water, Distance from WPP, Toilet, Pig, Cats, Ducks	2690.9	6.2
Sex, Age, Density, Tap water, Water truck, Tube well/Hand pump, Open well, Lake/Pond, Drinking water, Boil water, Distance from WPP, Toilet, Pig, Cats	2689.2	4.5
Sex, Age, Density, Tap water, Water truck, Tube well/Hand pump, Open well, Lake/Pond, Drinking water, Boil water, Distance from WPP, Toilet, Cats	2687.9	3.2
Sex, Age, Density, Tap water, Tube well/Hand pump, Open well, Lake/Pond, Drinking water, Boil water, Distance from WPP, Toilet, Cats	2686.7	2.0
Sex, Age, Density, Tap water, Tube well/Hand pump, Lake/Pond, Drinking water, Boil water, Distance from WPP, Toilet, Cats	2686.3	1.6
Sex, Age, Density, Tap water, Tube well/Hand pump, Drinking water, Boil water, Distance from WPP, Toilet, Cats	2685.3	0.6
Sex, Age, Tap water, Tube well/Hand pump, Drinking water, Boil water, Distance from WPP, Toilet, Cats	2685.0	0.3
Age, Tap water, Tube well/Hand pump, Drinking water, Boil water, Distance from WPP, Toilet, Cats	***2684.7***	0

Table S5. Number of diarrheal cases and Temperature and Rainfall amount in Nam Dinh province.

Year	Month	Number of diarrheal cases	Temperature max (℃)	Temperature average (℃)	Temperature minimum (℃)	Average Rainfall Amount (mm)	Rainy Days
2014	Oct	4	30	28	23	1	3
2014	Nov	37	26	24	21	1.05	5
2014	Dec	84	20	18	15	0.16	1
2014	Jan	40	21	19	16	0.66	4
2014	Feb	5	22	20	17	0.48	1
2014	Mar	28	24	22	20	1.36	13
2014	Apr	85	29	27	22	1.42	8
2014	May	77	36	33	28	5.24	9
2014	Jun	74	36	33	29	7.31	9
2014	Jul	76	33	31	27	3.31	9
2014	Aug	60	33	31	26	4.43	12
2014	Sep	97	32	30	26	6.96	18
2015	Oct	102	30	28	23	4.19	11
2015	Nov	31	27	25	22	4.07	16

Collection period is from 27 Oct 2014 to 16 Nov 2015. (Total cases were 800 cases.)

The routine visit was stopped for two weeks for the TET holidays in Vietnam in February 2015.

Temperature and Rainfall amount data is from World weather online (https://www.worldweatheronline.com/nam-dinh-weather-averages/vn.aspx)
